# FragMIL: A Fragment-Token Multiple-Instance Learning Framework for Cancer Detection from Cell-Free DNA Fragmentomics

**DOI:** 10.3390/cancers18203389

**Published:** 2026-10-20

**Authors:** Tien-Dung Bui, Yung-Feng Lin, Mai-Hanh Nguyen, Shih-Feng Tsai, Nguyen-Quoc-Khanh Le, Teh-Ying Chou

**Affiliations:** 1 International Ph.D. Program in Medicine, College of Medicine, Taipei Medical University, Taipei 110, Taiwan; d142113021@tmu.edu.tw; 2 Department of Pathology and Laboratory Medicine, Shin Kong Wu Ho-Su Memorial Hospital, Taipei 111, Taiwan; 3 AIBioMed Lab, Taipei Medical University, Taipei 110, Taiwan; nguyenmaihanh@vmmu.edu.vn; 4 Department of Internal Medicine, Taipei Medical University Hospital, Taipei Medical University, Taipei 110, Taiwan; 235009@h.tmu.edu.tw; 5 Taipei Cancer Center, Taipei Medical University Hospital, Taipei Medical University, Taipei 110, Taiwan; 6 Department of Pathology, 103 Military Hospital, Hanoi 100000, Vietnam; 7 Institute of Molecular and Genomic Medicine, National Health Research Institutes, Miaoli 350, Taiwan; petsai@nhri.edu.tw; 8 In-Service Master Program in Artificial Intelligence in Medicine, College of Medicine, Taipei Medical University, Taipei 110, Taiwan; 9 Translational Imaging Research Center, Taipei Medical University Hospital, Taipei 110, Taiwan; 10 Graduate Institute of Clinical Medicine, College of Medicine, Taipei Medical University, Taipei 110, Taiwan

**Keywords:** cell-free DNA, fragmentomics, liquid biopsy, multiple-instance learning, deep learning, cancer detection

## Abstract

**Background/Objectives**: Cell-free DNA (cfDNA) fragmentomics from low-coverage whole-genome sequencing (lcWGS) is a promising approach for non-invasive cancer detection. However, most existing methods summarize millions of cfDNA fragments into handcrafted sample-level features before classification, potentially discarding informative patterns carried by individual molecules. We aimed to develop and benchmark a framework that learns directly from fragment-level signals. **Methods**: We developed FragMIL, a fragment-token multiple-instance learning framework that represents each cfDNA molecule as a learnable token integrating end-motif, genomic-position, and fragment-length information, and aggregates thousands of fragment tokens into a patient-level cancer score. FragMIL was evaluated on 934 samples from three public lcWGS cohorts (pan-cancer, lung cancer, and hepatocellular carcinoma) using five repeated subject-level splits, a 288-configuration design-space benchmark, and comparison with four established fragmentomic approaches (coverage, DELFI, end motif, and fragment size). **Results**: FragMIL achieved the highest mean areas under the receiver operating characteristic curve (AUROCs) in every cohort, with 0.963 ± 0.009, 0.802 ± 0.009, and 0.910 ± 0.015 in the DELFI/Cristiano 2019, LUCAS/Mathios 2021, and Jiang 2015 cohorts, respectively. End-motif encoding provided the strongest standalone discriminative signal, whereas the optimal fragment pool was cohort-dependent, with open chromatin region fragments performing best for pan-cancer detection and tumor-enriched fragments for disease-specific cohorts. Performance was retained across cancer stages and cancer types. **Conclusions**: Preserving molecule-level fragmentomic information improves cancer detection, supporting fragment-token learning as a promising framework for next-generation cfDNA fragmentomics.

## Simple Summary

Blood contains small pieces of DNA released from cells throughout the body, including cancer cells. How these DNA pieces are cut—their length, the sequence at their ends, and where they come from in the genome—differs between people with and without cancer and can be measured with low-cost, low-coverage whole-genome sequencing. Existing tests usually condense millions of DNA pieces into a small set of average measurements, which may lose useful information. We developed FragMIL, an artificial intelligence approach that learns directly from thousands of individual DNA pieces per person and combines them into a single cancer score. Using public data from 934 individuals in three studies covering multiple cancer types, lung cancer, and liver cancer, FragMIL detected cancer more accurately than four established methods and remained informative across cancer stages and types. With prospective clinical validation, this approach could help improve blood-based cancer detection.

## 1. Introduction

Early and accurate cancer detection remains a central challenge in oncology, where stage at diagnosis is a primary determinant of survival [1,2]. Cell-free DNA (cfDNA) recovered from plasma offers a non-invasive window into tumor biology [3,4], and low-coverage whole-genome sequencing (lcWGS) has emerged as a practical platform for cfDNA analysis because it interrogates the entire genome at relatively low cost [5,6]. Beyond somatic mutations, cfDNA carries fragmentation patterns shaped by nucleosome positioning, chromatin accessibility, nuclease activity, and the relative contributions of different tissues to plasma DNA [7,8,9]. These patterns, collectively termed cfDNA fragmentomics, encode cancer-associated signals that can be extracted from lcWGS data without requiring deep sequencing or prior knowledge of tumor genotype, making fragmentomics an attractive basis for broad cancer detection [5,10,11].

The biological information captured by cfDNA fragmentomics spans several complementary layers. Fragment length and nucleosomal positioning reflect the chromatin state of the cell of origin; tumor-derived fragments are often enriched in shorter size ranges, and size-selected or tumor-enriched fragment subsets can increase the cancer signal [8,12]. The 5′ end-sequence motif of each fragment is shaped by nuclease cleavage preferences and carries tissue- and tumor-associated patterns that differ from healthy plasma DNA [10,13,14]. Genomic position provides a third layer: fragment endpoints near transcription start sites, within open chromatin regions, and across regulatory or gene-associated regions reflect the underlying chromatin accessibility and regulatory landscape [9,15,16,17]. These three layers—size, end motif, and genomic position—represent distinct but complementary views of the molecular information contained in each individual cfDNA fragment.

Prior machine-learning approaches to cfDNA fragmentomics have leveraged these signals by collapsing millions of fragments from each patient into fixed, sample-level summary vectors before classification. Averaging over all fragments dilutes the signal of tumor-derived fragments, which are a small fraction of cfDNA, particularly in early-stage disease [18]. Separate summaries of fragment length, end motif and genomic position also discard how these features co-occur on a single molecule. Three main strategies have been used. The first computes genome-wide aggregate statistics, such as end-motif frequencies across all fragments or fragment-size distribution histograms, and uses the resulting compact vectors as classifier input [5,10]. The second divides the genome into bins, typically at a resolution of 5 Mb or finer, and computes per-bin coverage, short-to-long ratios, or protection-related scores, producing high-dimensional but still sample-level matrices [5,8]. The third focuses on biologically defined regions of interest, including transcription start site windows, open chromatin regions, promoter-proximal regions, or other regulatory contexts, to capture region-specific fragmentation signals [9,16,19]. More recent integrative approaches have combined multiple fragmentomic feature families with ensemble or neural-network models, including integrated fragmentation patterns [19], early–late fusion of 13 feature spaces [20], a transformer model of end motifs [21] and frequency-domain end-motif models [22], to improve cancer detection [19,20]. These approaches have shown strong diagnostic performance, but they classify predefined sample-level descriptors rather than individual fragments. Only a few recent models learn directly from fragments, including a multiple-instance model for bisulfite-sequencing reads (DECIDIA) and a language model of randomly sampled cfDNA fragments (Fragmentia-AI) [23,24,25]. As a result, motif, size, and positional signals are usually summarized separately and then merged at the patient level, rather than being modeled jointly within the same individual cfDNA molecule before aggregation.

Recent advances in deep learning provide an opportunity to model cfDNA fragmentation at single-molecule resolution rather than through sample-level summaries. Attention-based multiple-instance learning, which encodes individual measurements as instances and learns to aggregate them into a sample-level prediction, has recently demonstrated this for sparse genomic data: Anaya et al. encoded individual somatic mutations for tumor-type and microsatellite-status classification and proposed extending the approach to circulating DNA fragments [26]. Building on this paradigm, we developed FragMIL, a fragment-token multiple-instance learning (MIL) framework for lcWGS cfDNA cancer detection [27,28], in which each cfDNA molecule is encoded as a token using paired-end endpoint motifs with a strand indicator, endpoint-aware genomic position, and patient-normalized fragment length, and thousands of fragment embeddings are aggregated to generate a patient-level cancer score. We applied this framework to three public lcWGS cfDNA cohorts to test whether fragment-level representation learning can improve cancer detection over established fragmentomic features.

## 2. Materials and Methods

### 2.1. Datasets

We analyzed 934 samples from three public cfDNA low-coverage whole-genome sequencing (lcWGS) cohorts. Of 1013 source samples, 79 were excluded: one healthy DELFI/Cristiano sample without a usable fragment file and 78 LUCAS/Mathios individuals without lung cancer at baseline who had another cancer or a prior or later lung cancer. The DELFI/Cristiano 2019 cohort [5] is a pan-cancer cfDNA dataset including eight cancer types and healthy controls. The LUCAS/Mathios 2021 cohort [6] contains lung cancer samples and non-cancer controls. The Jiang 2015 liver disease cohort [29] contains hepatocellular carcinoma (HCC), hepatitis B, cirrhosis and healthy control samples, and was used to evaluate cancer detection in a liver-disease screening context. DELFI/Cristiano 2019 and Jiang 2015 data were obtained from the FinaleDB database [30], whereas LUCAS/Mathios 2021 data were obtained from the European Genome-phenome Archive [31] under Dataset ID EGAD00001007796. Fragment files of DELFI/Cristiano 2019 and Jiang 2015 were used as provided by FinaleDB, and those of LUCAS/Mathios 2021 were generated from the deposited BAM files (Supplementary Methods S1).

Cancer-versus-non-cancer classification was performed in all three cohorts at the patient level. Stage-specific analyses were performed in DELFI/Cristiano 2019 and LUCAS/Mathios 2021 because stage metadata were available in these cohorts. Cancer-type-specific analyses were performed only in DELFI/Cristiano 2019 because this was the multi-cancer cohort. Detailed sample counts, cancer-type composition and metadata are provided in Table 1 and Supplementary Table S1, and the age, sex and other available patient characteristics by class are provided in Supplementary Table S2.

### 2.2. Fragment-Token Representation and Patient-Level Aggregation

**Figure 1:**
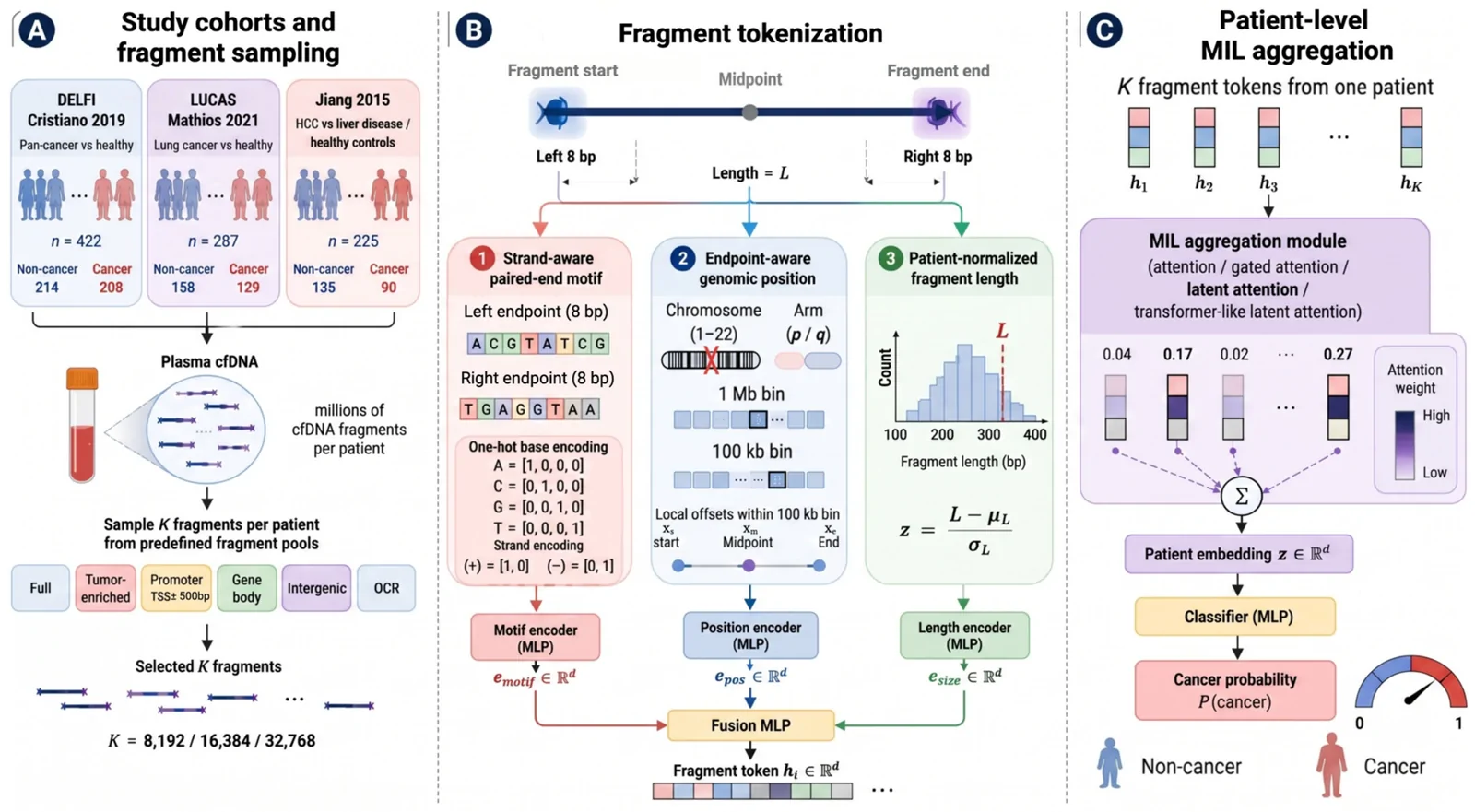
Study workflow. (**A**) Study cohorts and fragment sampling: cfDNA fragments from three public lcWGS cohorts were used for cancer classification. For each patient, K fragments were sampled from predefined fragment pools, including full-fragment, tumor-enriched, promoter, gene-body, intergenic and open chromatin region (OCR) pools. (**B**) Fragment tokenization: each cfDNA fragment is encoded into a fragment token via three input branches—paired-end endpoint motifs with a strand indicator, endpoint-aware genomic position, and patient-normalized fragment length. (**C**) Patient-level MIL aggregation: K fragment tokens from each patient were aggregated by a multiple-instance learning module to generate a patient embedding, which was passed through a classifier to produce a patient-level cancer probability.

FragMIL represents each patient as a bag of cfDNA fragment tokens (Figure 1). A fragment encoder embeds the end motif, genomic position and length of each fragment, an MIL aggregation module combines the embeddings of the bag into a patient embedding, and a classifier outputs the probability of cancer. Only the patient-level label is used for training. Raw fragment files were preprocessed to ensure that the retained fragments had high mapping-quality scores, valid genomic coordinates and a standardized structure suitable for downstream tokenization. Only autosomal fragments (chr1–chr22) with valid coordinates, positive length, a valid strand annotation and mapping quality ≥ 30 were retained; fragments with ambiguous reference bases at either 8 bp endpoint were removed; fragments overlapping UCSC hg19 assembly gaps or ENCODE blacklist (v2) regions were masked out; and PCR duplicates had been removed when the fragment files were generated from the aligned reads, with no additional deduplication applied. Details of fragment preprocessing, coordinate conventions, endpoint extraction, quality filtering and bad-region masking are provided in the Supplementary Methods.

Each cfDNA molecule was encoded as a fragment token with three information branches: paired-end motif, genomic position and fragment length. The paired-end motif branch encoded the 8 bp sequence at the left endpoint and the 8 bp sequence at the right endpoint of each fragment. During training, each nucleotide was converted to a four-dimensional one-hot vector (A = [1, 0, 0, 0]; C = [0, 1, 0, 0]; G = [0, 0, 1, 0]; T = [0, 0, 0, 1]). For a patient bag containing K fragments, the left-end and right-end sequences were represented as tensors of shape [K, 8, 4]. The two endpoint tensors were flattened so that the 16 endpoint bases contributed 16 × 4 = 64 motif values per fragment. Fragment strand was encoded as a two-dimensional one-hot vector (+strand = [1, 0]; −strand = [0, 1]). The 64 endpoint motif values and the 2 strand values were concatenated to form a 66-dimensional motif input for each fragment. Endpoint sequences were not reverse-complemented or swapped according to strand; instead, strand identity was supplied directly to the model. A multilayer perceptron transformed this 66-dimensional input into a motif embedding.

The genomic-position branch encoded both broad and local genomic location. The chromosome identity, chromosome arm, 1 Mb genomic bin and 100 kb genomic bin were treated as categorical variables and transformed into learnable embeddings. To preserve local position within each 100 kb bin, we included the normalized coordinates of the fragment start, fragment end and fragment midpoint. The categorical position embeddings and the three normalized local coordinates were concatenated and passed through a position encoder.

The fragment-length branch encoded size as a patient-normalized fragment length. For each patient, the mean and standard deviation of fragment length were computed from all high-quality autosomal fragments, independent of the fragment pool being analyzed. The patient-normalized length z-score was then passed through a size encoder.

The motif, position and size embeddings were fused into a fragment-level embedding. To evaluate the contribution of each information source, four input modes were compared: end-motif branch, position branch, size branch and three-branch model. In the three-branch model, all three embeddings were concatenated and passed through a fusion multilayer perceptron to generate the final fragment embedding.

Each patient was represented as a bag of K fragment embeddings. Patient-level prediction was performed using multiple-instance learning, which aggregated the K fragment embeddings into a patient embedding and then transformed the patient embedding into a cancer probability. Four aggregation modules were evaluated. Attention MIL [27] assigns a learnable score to each fragment embedding, normalizes the scores across the bag with a softmax and sums the embeddings weighted by these attention weights. Gated attention MIL computes the score from the element-wise product of a tanh and a sigmoid transformation of the embedding, which allows non-linear interactions between embedding features. In latent attention [32], a small set of learnable latent vectors attends to all fragment embeddings by scaled dot-product attention, and the outputs are averaged into the patient embedding. Transformer-like latent attention [33] additionally applies self-attention among the latent vectors, feed-forward layers and residual connections. All four modules are invariant to the order of the fragments. Detailed equations are provided in the Supplementary Methods.

### 2.3. Fragment Sampling, Fragment Pools and Benchmark Design

Because each cfDNA sample contains a large number of fragments, patient bags were constructed by sampling a fixed number of fragments per patient. Three fragment budgets were evaluated: K = 8192, K = 16,384 and K = 32,768. During training, one K-fragment bag was sampled for each patient at each epoch, and training was capped at 100 epochs. Fragments were sampled without replacement within each bag when sufficient fragments were available, but the same fragment could be sampled again in a different epoch or test-time bag.

Six fragment pools were evaluated. The full-fragment pool sampled uniformly from all high-quality autosomal fragments and served as the genome-wide random-fragment setting. The tumor-enriched fragment pool selected fragments using a size criterion and a nucleosome-overlap criterion, designed to enrich fragments with tumor-associated nucleosomal patterns. Four additional region-restricted pools were defined by midpoint overlap with promoter, gene-body, intergenic and open chromatin region (OCR) intervals. These region-restricted pools were included because previous cfDNA fragmentomics studies have shown that transcription start site (TSS)-associated coverage, open-chromatin fragmentation and region-specific fragmentation patterns can carry cancer- or tissue-related information [9,16,19,20]. Detailed definitions of fragment pools, genomic regions, nucleosome-protected regions and fragment assignment rules are provided in the Supplementary Methods.

The full benchmark grid crossed six fragment pools, three fragment budgets, four input modes and four MIL aggregation modules, giving 288 fragment-token MIL configurations per cohort. This design allowed us to evaluate both the best-performing configuration in each cohort and the marginal contribution of fragment pool, fragment budget, token information source and aggregation module.

### 2.4. Model Training, Baseline Comparison and Statistical Evaluation

All models were trained for binary cancer-versus-non-cancer classification using subject-level train, validation and test splits. Each cohort was split approximately 70%/15%/15% into training, validation and test sets, and this procedure was repeated five times with independent random seeds. The patient identifiers of every split are provided in Supplementary Table S3. The same five split files were used for all fragment-token MIL configurations and all baseline models. No fragments from the same patient were shared across partitions.

Fragment-token MIL models were trained using distributed data parallel training across 8 NVIDIA Tesla V100-SXM2 16 GB GPUs (NVIDIA Corporation, Santa Clara, CA, USA), with one patient bag processed per GPU and an effective batch size of 8 patients. Models were optimized with learning rate of 1 × 10^−4^, weight decay of 1 × 10^−3^ and dropout of 0.3. Training used binary cross-entropy loss and early stopping based on validation loss, with patience of 20 epochs, minimum delta of 0.0005 and at least 40 epochs before early stopping was allowed. Training was capped at 100 epochs. Mixed-precision training was used where supported. Fragment-token MIL models were implemented in Python 3.10 with PyTorch 2.7.1 (CUDA build 12.6), and the baseline logistic-regression models were implemented with scikit-learn 1.7.2. Validation used five presampled bags per epoch, and test-time evaluation averaged predictions over 100 presampled bags for each patient.

We compared fragment-token MIL with four established fragmentomic feature groups: coverage, DELFI, end motif and fragment size (fragment-size distribution, FSD). These baselines were selected to represent widely used coverage-, genome-bin ratio-, end motif- and size-based cfDNA fragmentomic signals reported in previous cancer detection studies [5,10,16,19]. Each baseline feature group was evaluated separately using L2-regularized logistic regression on standardized features. Regularization strength was selected using the validation partition of each repeat. Support vector machine, random forest and multilayer perceptron classifiers **(scikit-learn 1.7.2)** and XGBoost classifiers **(XGBoost 3.1.1)** were also evaluated on the same features and splits (Supplementary Table S4). Detailed baseline feature definitions and implementation are provided in the Supplementary Methods [5,6,9,10,12,16,19,27,30,32,33,34,35,36,37,38,39].

The primary evaluation metrics were the area under the receiver operating characteristic curve (AUROC) and the area under the precision–recall curve (AUPRC). Each repeat produced one patient-level cancer probability per test sample. For fragment-token MIL, predictions from the 100 test-time bags were averaged to generate one patient-level score; individual bags were not treated as independent samples. Performance was summarized as mean ± standard deviation across the five repeated splits. Stage-stratified and cancer-type-stratified analyses were computed using the same patient-level prediction scores. Sensitivity, specificity, and positive and negative predictive values (PPV and NPV) were calculated at target specificities of 90% and 95% (Supplementary Table S5). Calibration was assessed with the Brier score, expected calibration error, and calibration slope and intercept (Supplementary Table S6).

Generative AI tools (ChatGPT, GPT-5.5; OpenAI, San Francisco, CA, USA; and Claude, Opus 4.7; Anthropic, San Francisco, CA, USA) were used for language editing and the AI features of BioRender (Science Suite Inc., Toronto, ON, Canada) for the schematic in Figure 1; they were not used for study design, data analysis or interpretation.

## 3. Results

### 3.1. Fragment-Token MIL Framework for cfDNA Cancer Detection

We developed FragMIL, a fragment-token MIL framework for cancer detection from low-coverage whole-genome cfDNA sequencing. Instead of summarizing each sample into fixed handcrafted fragmentomic features, the framework treats each cfDNA molecule as an individual learnable token and aggregates thousands of fragment tokens into a patient-level cancer probability. The approach was evaluated across three public lcWGS cfDNA cohorts comprising 934 samples included in the analysis: the DELFI/Cristiano 2019 pan-cancer cohort [5], the LUCAS/Mathios 2021 lung cancer cohort [6], and the Jiang 2015 liver disease cohort [29] (Figure 1A, Table 1, Supplementary Table S1). The median number of raw fragments per sample was 36.6, 31.3 and 30.4 million in DELFI/Cristiano 2019, LUCAS/Mathios 2021 and Jiang 2015, respectively.

Each fragment token encoded three complementary molecular contexts: paired-end endpoint sequence motifs with a strand indicator, endpoint-aware genomic position, and patient-normalized fragment length (Figure 1B). These token-level representations were sampled from genome-wide, tumor-enriched, and region-restricted fragment pools and then passed through deep MIL aggregation to generate patient-level predictions (Figure 1C). This design allowed the model to learn cancer-associated fragmentomic patterns directly from fragment-level heterogeneity while preserving the ability to test which input branches, fragment pools, fragment budgets, and MIL aggregators contributed most to performance.

### 3.2. Fragment-Token MIL Improves Cancer Detection Across Three Public lcWGS Cohorts

**Figure 2:**
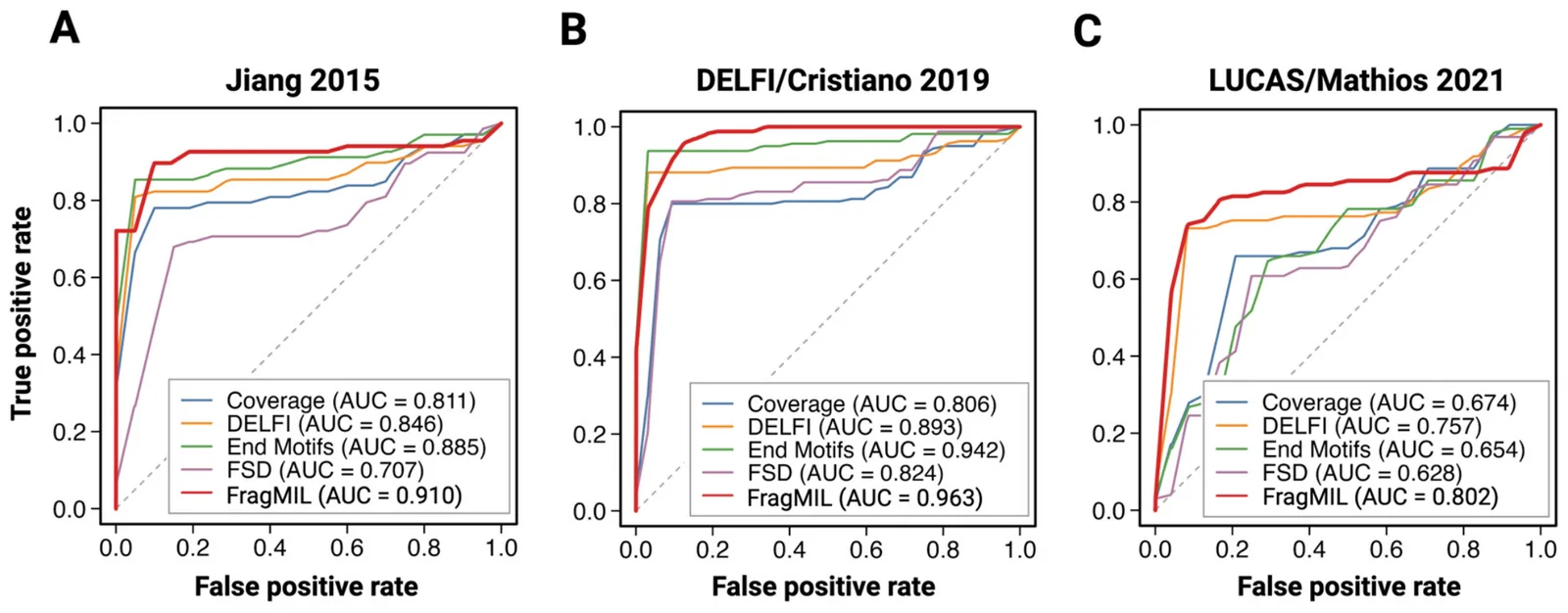
Cancer detection ROC curves for fragment-token MIL and fragmentomic baseline feature groups across three cohorts. ROC curves comparing the best fragment-token MIL configuration with four established cfDNA fragmentomic features (coverage, DELFI, end motif, and FSD) for (**A**) Jiang 2015 (HCC versus non-cancer controls), (**B**) DELFI/Cristiano 2019 (pan-cancer versus healthy controls), and (**C**) LUCAS/Mathios 2021 (lung cancer versus non-cancer controls).

The best fragment-token MIL models achieved strong cancer-versus-non-cancer discrimination across all three cohorts, with mean test AUROCs of 0.963 ± 0.009 in DELFI/Cristiano 2019, 0.802 ± 0.009 in LUCAS/Mathios 2021, and 0.910 ± 0.015 in Jiang 2015 (Figure 2, Table 2). The corresponding AUPRCs were 0.955 ± 0.015, 0.769 ± 0.016, and 0.925 ± 0.008, respectively. The best-performing configurations differed by cohort and are summarized in Supplementary Table S7.

To determine whether fragment-token learning improved cancer detection beyond conventional sample-level fragmentomics, we compared the best fragment-token MIL model in each cohort with four established baseline feature groups: coverage, DELFI, end motif, and fragment size. These baselines represent commonly used cfDNA fragmentomic signals previously reported in cancer detection studies [5,6,9,10]. All baseline models were evaluated on the same five repeated subject-level splits as the fragment-token MIL models.

Fragment-token MIL had a higher mean AUROC than the strongest handcrafted baseline in every cohort (Figure 2, Table 2). The AUROC was 0.963 ± 0.009 versus 0.942 ± 0.026 for end motif in DELFI/Cristiano 2019, 0.802 ± 0.009 versus 0.757 ± 0.052 for DELFI in LUCAS/Mathios 2021, and 0.910 ± 0.015 versus 0.885 ± 0.035 for end motif in Jiang 2015 (paired differences with 95% confidence intervals are given in Supplementary Table S9). The mean AUPRC was also highest for fragment-token MIL in all three cohorts (Table 2, Supplementary Figure S1). Four additional classifiers (support vector machine, random forest, XGBoost and multilayer perceptron) exceeded logistic regression by at most 0.019 AUROC, and FragMIL had a higher mean AUROC than every baseline classifier (Supplementary Table S4). At target specificities of 90% and 95%, FragMIL reached sensitivities of 0.91 and 0.79 in DELFI/Cristiano 2019, 0.74 and 0.57 in LUCAS/Mathios 2021, and 0.90 and 0.72 in Jiang 2015; at 95% specificity, the strongest baseline had a higher sensitivity in DELFI/Cristiano 2019 and Jiang 2015 (Supplementary Table S5).

### 3.3. Systematic Design-Space Benchmarking Identifies Informative Fragment-Token Components

**Figure 3:**
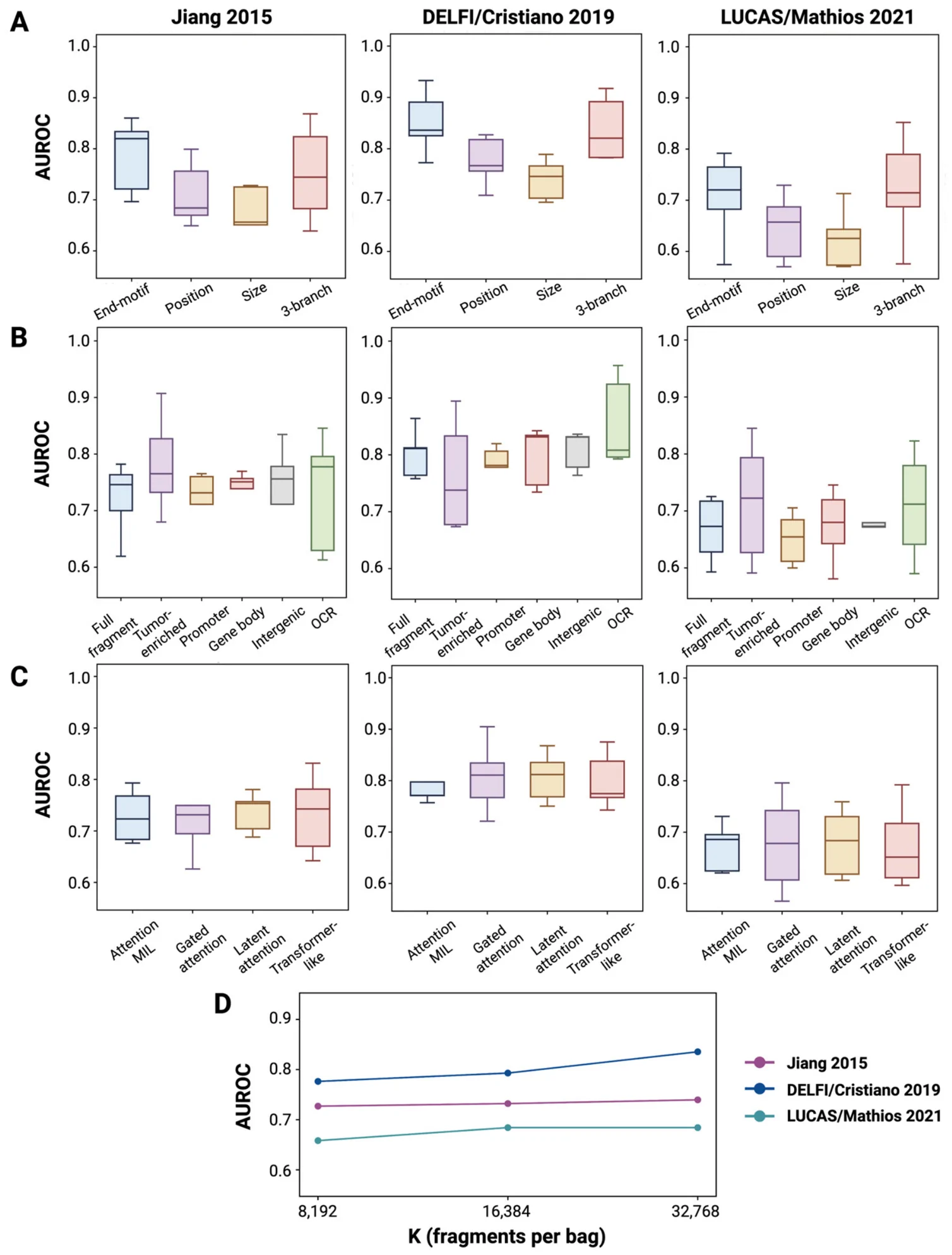
Fragment-token MIL performance across fragment pools, branch modes, aggregation modules, and fragment budgets. (**A**) Branch mode (input branches used to encode each cfDNA fragment). (**B**) Fragment pool (genome-wide, tumor-enriched, or region-restricted cfDNA fragment subset). (**C**) Aggregation module (MIL architecture used to aggregate fragment embeddings into a patient-level score). (**D**) Fragment budget (K fragments sampled per patient bag at each training epoch). OCR, open chromatin regions.

We next investigated which components of the fragment-token framework carried the most discriminative information. Rather than evaluating only one optimized model per component, we summarized marginal AUROC across the full 288-configuration benchmark grid. This allowed branch modes, fragment pools, fragment budgets, and MIL aggregators to be compared under the same repeated-split evaluation framework (Figure 3, Supplementary Figure S2, Supplementary Table S8).

Each fragment token was designed to encode three major sources of molecular information: paired-end sequence motifs, genomic-position context, and patient-normalized fragment length. We therefore compared models using each branch alone with a combined three-branch model to determine which information source contributed most strongly to cancer detection (Figure 3A). End-motif information provided the most consistent standalone signal. The end-motif branch achieved the highest marginal AUROC in DELFI/Cristiano 2019 (0.852 ± 0.062) and Jiang 2015 (0.786 ± 0.073). In LUCAS/Mathios 2021, the combined three-branch model performed best (0.724 ± 0.105), followed by the end-motif branch (0.707 ± 0.085). Thus, end-motif encoding was the strongest single-branch representation overall, whereas multi-branch fusion provided additional benefit in the lung cancer cohort.

Fragment pool selection showed a cohort-dependent pattern, with no single pool performing best across all cohorts (Figure 3B). In the pan-cancer DELFI/Cristiano 2019 cohort, the OCR pool reached the highest mean marginal AUROC (0.856 ± 0.079), whereas the tumor-enriched pool ranked highest in both disease-focused cohorts (0.716 ± 0.108 in LUCAS/Mathios 2021 and 0.782 ± 0.088 in Jiang 2015). This cohort-dependent pattern suggests that different fragment pools may enrich different layers of the cfDNA signal: OCRs may capture regulatory-region and tissue-of-origin information across heterogeneous cancer types, whereas the tumor-enriched pool may concentrate nucleosome- and size-associated tumor signals in more disease-specific settings.

We next compared four MIL aggregation modules: attention MIL, gated attention MIL, latent attention and transformer-like latent attention (Figure 3C). Aggregator choice produced smaller differences than branch mode or fragment pool. Across cohorts, the four aggregators showed broadly comparable marginal AUROCs, suggesting that the main performance drivers were the fragment-token representation and the selected fragment pool rather than a single aggregation architecture.

Finally, we evaluated whether increasing the number of fragment tokens per patient improved model performance (Figure 3D). Increasing K generally improved AUROC, but the benefit differed by cohort. Marginal AUROC increased from 0.776 to 0.793 and 0.836 in DELFI/Cristiano 2019, from 0.658 to 0.684 and 0.684 in LUCAS/Mathios 2021, and from 0.727 to 0.732 and 0.740 in Jiang 2015 for K = 8192, 16,384 and 32,768, respectively. Thus, larger fragment bags provided the clearest gain in the pan-cancer cohort, whereas performance plateaued earlier in the two more disease-focused cohorts. Together, the design-space benchmark indicates that end-motif encoding was the most consistent determinant of performance, whereas the best-performing fragment pool and fragment budget were cohort-dependent.

### 3.4. Model Performance Remains Informative Across Cancer Stages and Cancer Types

**Figure 4:**
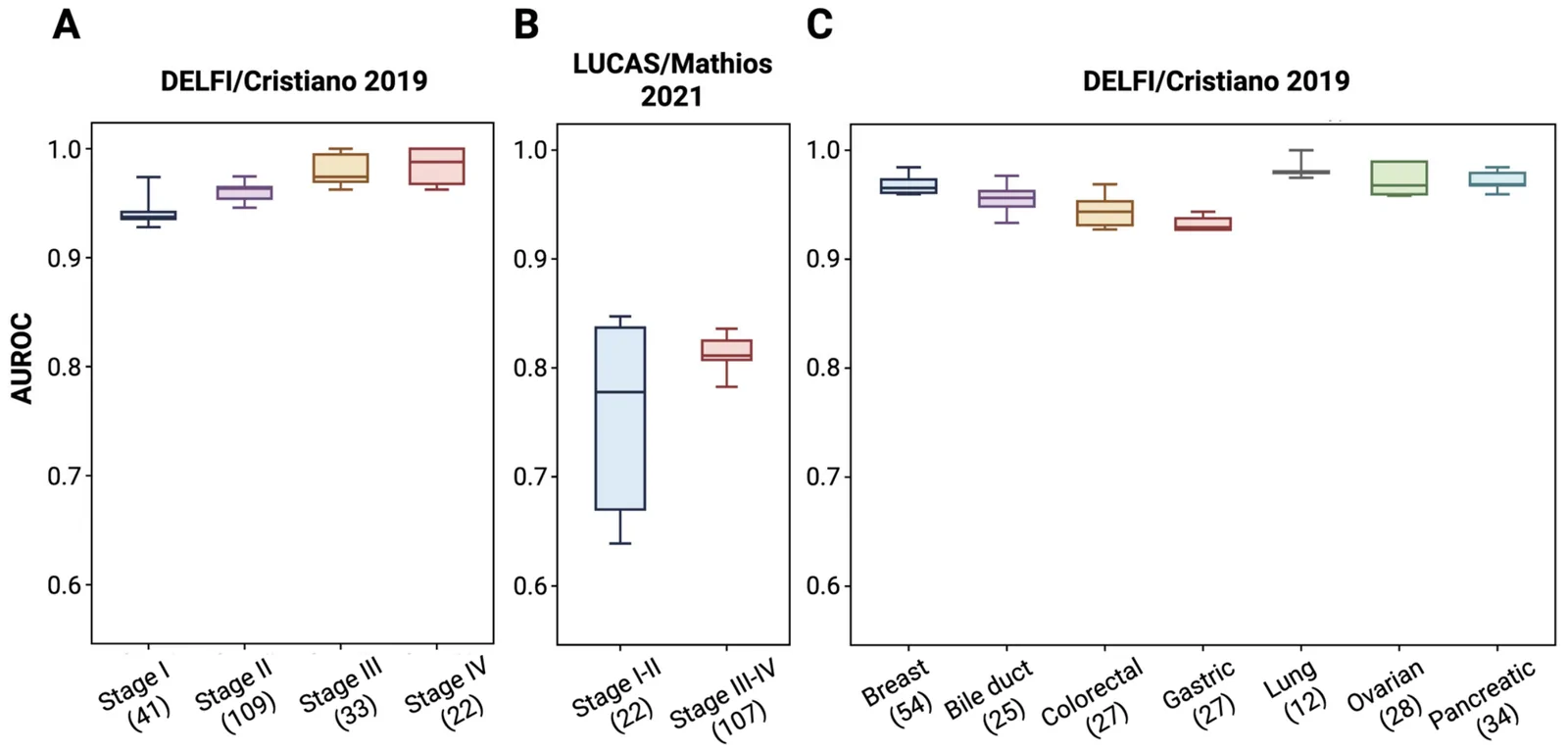
Stage-specific and cancer-type-specific performance of fragment-token MIL. (**A**) Stage-specific cancer-versus-healthy AUROC in the DELFI/Cristiano 2019 cohort (Stage I–IV). (**B**) Stage-specific cancer-versus-non-cancer AUROC in the LUCAS/Mathios 2021 cohort (Stage I–II and Stage III–IV). (**C**) Cancer-type-specific cancer-versus-healthy AUROC in the DELFI/Cristiano 2019 cohort across seven cancer types.

Because early-stage disease and different tumor types can release different amounts and forms of tumor-derived cfDNA [18], we next evaluated whether fragment-token MIL retained discriminative performance across cancer stages and cancer types (Figure 4). This analysis was performed in cohorts with available subgroup metadata and used subgroup-specific cancer-versus-control comparisons within the repeated evaluation framework.

In DELFI/Cristiano 2019, model performance increased with cancer stage (Figure 4A; Supplementary Table S10). The best fragment-token MIL model achieved AUROC 0.943 ± 0.018 for Stage I disease, 0.961 ± 0.011 for Stage II, 0.980 ± 0.016 for Stage III, and 0.984 ± 0.018 for Stage IV. A similar pattern was observed in LUCAS/Mathios 2021 (Figure 4B). Because Stage II contained only seven cancer samples, stages were grouped into Stage I–II and Stage III–IV. The Stage I–II group achieved AUROC 0.754 ± 0.095, whereas the Stage III–IV group achieved 0.812 ± 0.020. This stage-dependent pattern is consistent with the expected increase in the tumor-derived cfDNA signal with increasing disease burden [18].

We also evaluated cancer-type-specific discrimination in the DELFI/Cristiano 2019 cohort (Figure 4C). Duodenal cancer was excluded from this analysis because only one usable sample was available. Across the seven retained cancer types, AUROC ranged from 0.933 to 0.983, with the highest performance observed for lung cancer (0.983 ± 0.010) and the lowest for gastric cancer (0.933 ± 0.007). Differences across cancer types are expected because tumor burden, tissue of origin, chromatin organization, and cfDNA shedding patterns vary across malignancies.

Under the unified default configuration (Supplementary Table S11), the 1% of fragments with the highest attention weights were enriched for 100–150 bp fragments in correctly detected cancers (median fold enrichment, 1.44–1.55) more than in correctly classified non-cancer controls (1.17–1.18; *p* < 0.001 in all cohorts) and were depleted of CCCA end motifs, whereas CCCG motifs showed no enrichment (Supplementary Table S12).

## 4. Discussion

cfDNA fragmentomics has established multiple cancer-associated signals, including genome-wide fragmentation patterns, DELFI short-to-long fragment ratios, nucleosome footprints, 5′ end motifs, open-chromatin fragmentation, and TSS-associated coverage patterns [5,8,9,10,12,16]. These approaches have demonstrated the diagnostic value of cfDNA fragmentation, but most analytical pipelines first collapse millions of fragments into sample-level feature summaries before classification. Our study takes a different approach by shifting the unit of analysis from the sample-level feature matrix to the individual cfDNA molecule. Each fragment is represented as a learnable token that jointly encodes paired-end motif, genomic-position context, and patient-normalized fragment length into a single vector before patient-level aggregation. This design allows information carried by the same molecule to be modeled together, rather than being separated into independent handcrafted features such as motif frequencies, size histograms, or regional coverage scores. In this approach, fragment-token learning provides a molecule-level extension of conventional cfDNA fragmentomics. Unlike recent fragment-level sequence models such as Fragmentia-AI and DECIDIA [23,24], which learn representations directly from read sequences, FragMIL encodes endpoint motif, genomic position and fragment length in separate, interpretable branches, which makes the contribution of each information source measurable but does not capture the internal sequence of the fragment. For sample-level multimodal models evaluated on the same cohorts, Lu et al. [20] reported cross-validation AUCs of 0.972, 0.816 and 0.972 for ELSM and 0.950, 0.764 and 0.937 for the model of Moldovan et al. [40] in DELFI/Cristiano 2019, LUCAS/Mathios 2021 and Jiang 2015, respectively, compared with 0.963, 0.802 and 0.910 for FragMIL. These values are not directly comparable, because Lu et al. used 10-fold cross-validation with their own feature extraction and sample sets, whereas our estimates derive from five repeated 70%/15%/15% splits with held-out test sets and from the best of 288 configurations per cohort (Table 2).

A major practical challenge is that each lcWGS sample contains millions to tens of millions of cfDNA fragments, making exhaustive fragment-level deep learning computationally infeasible. Prior cfDNA fragment-end analyses showed that cancer-discriminative fragment-end composition remains detectable from low-coverage whole-genome sequencing even under strong in silico dilution and at low tumor fractions [40], indicating that some fragmentomic signals are sufficiently dense to be recovered from limited fragment subsets. Motivated by this, we used randomly sampled fragment bags, with K fragments per patient at each training step and resampling across 100 epochs, so that the model was exposed to multiple molecular subsets rather than a single fixed subset. This strategy makes fragment-token learning computationally tractable, but also means that performance depends on how many fragments are sampled and whether the sampled molecules represent the informative part of the fragmentome. In our benchmark, increasing K generally improved performance, but the gain from K = 16,384 to K = 32,768 was cohort-dependent. Marginal AUROC increased from 0.776 to 0.793 and 0.836 in DELFI/Cristiano 2019, from 0.658 to 0.684 and 0.684 in LUCAS/Mathios 2021, and from 0.727 to 0.732 and 0.740 in Jiang 2015 for K = 8192, 16,384, and 32,768, respectively (Figure 3D). These results suggest that larger fragment bags do not provide uniform gains across cohorts. K = 16,384 appeared sufficient in the disease-focused LUCAS and Jiang cohorts, where increasing to K = 32,768 produced little additional improvement. By contrast, marginal AUROC in DELFI/Cristiano 2019 continued to increase up to the largest K we tested; we did not evaluate larger fragment budgets owing to the computational cost of fragment-token training, and whether K > 32,768 yields further gains in this pan-cancer setting remains to be explored in future work. Sampling a subset of the available fragments is also the strategy of other fragment-level models, which used 3000 fragments per sample and epoch (Fragmentia-AI) or 1000 reads per sample (DECIDIA) [23,24]; the bags used here (8192–32,768 fragments) were larger.

Each fragment token contained three major information branches: paired-end motif, genomic position, and fragment length. Among these, the end-motif branch provided the most consistent standalone signal. This is consistent with previous studies showing that cfDNA fragment ends are biologically structured and can reflect nuclease activity, tissue contribution, and cancer-associated fragmentation processes [10,41]. In our benchmark, the end-motif branch was the strongest single branch and exceeded the combined three-branch representation in two of three cohorts (DELFI/Cristiano 2019 and Jiang 2015 in the marginal averages across the grid; LUCAS/Mathios 2021 and Jiang 2015 in the best configuration of each cohort). This does not imply that position and length are uninformative. Rather, it suggests that end-motif information was the most directly learnable signal under the current encoder and fusion design. Position and size features may require larger training cohorts, stronger regularization, or more adaptive fusion strategies to provide consistent incremental benefit. The LUCAS cohort, where the combined three-branch model had the higher marginal AUROC, supports this interpretation and indicates that multi-branch fusion can be useful when the cohort and model architecture allow complementary signals to be integrated effectively. This is also consistent with recent multimodal cfDNA frameworks such as ELSM, which emphasized that integrating multiple fragmentomic modalities can improve cancer detection but requires careful fusion in high-dimensional, small-sample settings [20].

Randomly sampling fragments from predefined genomic or biological regions also produced different performance patterns across cohorts. The tumor-enriched fragment pool was designed to enrich fragments with nucleosome- and size-associated tumor signal, whereas promoter, gene-body, intergenic, and OCR pools sampled distinct genomic contexts. Promoter and TSS-centered regions are supported by prior work showing that cfDNA coverage around TSSs reflects nucleosome occupancy and gene-expression-related chromatin structure [16]. OCR-based fragmentation is supported by studies showing that open chromatin regions carry tissue-of-origin and regulatory-region fragmentation information [9]. More broadly, systematic comparisons of cfDNA fragmentation patterns have shown that signals can be defined at different genomic scales and that integrating multiple fragmentation patterns can enhance cancer detection [19]. In our study, the best-performing pool was cohort-dependent: OCR ranked highest in the heterogeneous pan-cancer DELFI/Cristiano 2019 cohort, whereas the tumor-enriched pool ranked highest in the more disease-focused LUCAS lung cancer and Jiang HCC cohorts. This suggests that fragment selection may depend on tumor type, cohort composition, and the biological context captured by each region set, rather than following a single universal ranking.

Several limitations should be considered. First, performance and optimal model configuration were not fully consistent across cohorts, indicating that fragment-token learning remains sensitive to disease context, cohort composition, and technical workflow. This is expected in cfDNA fragmentomics, where feature quantification can be influenced by library preparation, sequencing protocol, and data-processing choices; Wang et al. recently showed substantial variation in fragmentomic features across nine library kits and ten processing routes [42]. Second, random K-fragment sampling may not always represent the full fragmentome, particularly when informative tumor-derived fragments are rare. Although resampling across 100 epochs reduces dependence on a single subset, it does not fully replace exhaustive fragment-level modeling. Third, the present framework was evaluated on retrospective public cohorts, and prospective validation using harmonized preprocessing will be needed to determine how robustly the approach generalizes across clinical and technical settings. In addition, each model was trained and tested within the same cohort, and cross-cohort validation was not performed. Fourth, this study is an in-domain benchmark in which each model was trained and tested within a single cohort. Cross-cohort validation was not performed because the cohorts differ in cancer types, control groups and data-processing pipelines, so the reported performance reflects within-cohort discrimination and requires confirmation in independent cohorts.

Overall, our framework showed stable performance across multiple public cohorts, cancer types, and disease stages. By encoding each cfDNA molecule as a fragment token before aggregation, this study opens a new deep-learning direction for maximizing the information contained in individual cfDNA fragments.

## 5. Conclusions

This study proposes FragMIL, a fragment-token multiple-instance learning framework for cfDNA cancer detection from lcWGS data. By encoding each cfDNA molecule as a learnable token and aggregating fragment-level embeddings into patient-level cancer scores, the framework provides a molecule-level extension of conventional fragmentomic analysis and improves detection across multiple public cohorts, cancer types and disease stages. These findings support fragment-token learning as a promising deep-learning strategy for maximizing information from individual cfDNA fragments. Future work will extend this approach beyond paired-end motifs by encoding the full nucleotide sequence of each fragment, enabling models to capture internal sequence composition, local motif structure, positional context and fragment-size information across the entire cfDNA molecule for more informative and interpretable cancer detection.

## Figures and Tables

**Table 1:** Cohort characteristics.

Cohort	*n*	Cancer (Label = 1), *n*	Non-Cancer (Label = 0), *n*	Stage Distribution
Jiang 2015	225	90 (HCC)	135 ^b^	Not available per sample ^c^
DELFI/Cristiano 2019	422	208 ^a^	214 (healthy)	Stage I: 41 Stage II: 109 Stage III: 33 Stage IV: 22
LUCAS/Mathios 2021	287	129 (lung cancer)	158 (healthy)	Stage I: 15 Stage II: 7 Stage III: 35 Stage IV: 72

^a^ Eight cancer types: Colorectal cancer (*n* = 27), breast cancer (*n* = 54), lung cancer (*n* = 12), ovarian cancer (*n* = 28), pancreatic cancer (*n* = 34), bile duct cancer (*n* = 25), gastric cancer (*n* = 27), duodenal cancer (*n* = 1). Duodenal cancer was excluded from cancer-type-specific analyses (single sample). ^b^ Non-cancer controls: Hepatitis B (*n* = 67), cirrhosis (*n* = 36), healthy (*n* = 32). The classification task was HCC versus all non-cancer controls (label = 0). ^c^ Stage information is not available at the per-sample level for the Jiang 2015 cohort. HCC, hepatocellular carcinoma. Stage was available for 205 of the 208 DELFI/Cristiano 2019 cancer samples; the remaining three were recorded as Stage 0 (*n* = 2) or Stage X (*n* = 1) and are not included in the stage distribution (Supplementary Table S1). The 158 non-cancer controls of LUCAS/Mathios 2021 comprised 91 healthy individuals and 67 patients with benign lung lesions.

**Table 2:** Cancer detection performance of fragment-token MIL and fragmentomic baseline feature groups across three cohorts.

Cohort	Method	AUROC, Mean ± SD [95% CI]	AUPRC, Mean ± SD [95% CI]
Jiang 2015	Coverage	0.811 ± 0.043 [0.732, 0.887]	0.804 ± 0.067 [0.735, 0.893]
	DELFI	0.846 ± 0.039 [0.769, 0.920]	0.821 ± 0.076 [0.758, 0.921]
	End motif	0.885 ± 0.035 [0.819, 0.950]	0.882 ± 0.042 [0.827, 0.945]
	Fragment size	0.707 ± 0.055 [0.616, 0.798]	0.635 ± 0.085 [0.577, 0.771]
	Best fragment-token MIL †	0.910 ± 0.015 [0.855, 0.962]	0.925 ± 0.008 [0.881, 0.966]
DELFI/Cristiano 2019	Coverage	0.806 ± 0.035 [0.754, 0.858]	0.792 ± 0.044 [0.747, 0.868]
	DELFI	0.893 ± 0.030 [0.848, 0.934]	0.899 ± 0.050 [0.860, 0.956]
	End motif	0.942 ± 0.026 [0.910, 0.974]	0.938 ± 0.035 [0.899, 0.978]
	Fragment size	0.824 ± 0.038 [0.768, 0.873]	0.794 ± 0.039 [0.742, 0.872]
	Best fragment-token MIL †	0.963 ± 0.009 [0.944, 0.981]	0.955 ± 0.015 [0.931, 0.980]
LUCAS/Mathios 2021	Coverage	0.674 ± 0.061 [0.598, 0.750]	0.612 ± 0.087 [0.569, 0.720]
	DELFI	0.757 ± 0.052 [0.683, 0.830]	0.726 ± 0.069 [0.669, 0.821]
	End motif	0.654 ± 0.064 [0.574, 0.733]	0.595 ± 0.086 [0.546, 0.692]
	Fragment size	0.628 ± 0.067 [0.553, 0.707]	0.568 ± 0.096 [0.530, 0.667]
	Best fragment-token MIL †	0.802 ± 0.009 [0.733, 0.870]	0.769 ± 0.016 [0.718, 0.872]

AUROC, area under the receiver operating characteristic curve; AUPRC, area under the precision–recall curve. † Selected from a 288-configuration benchmark grid; full rankings are provided in Supplementary Table S8. Baselines: L2-regularized logistic regression; the other classifiers are reported in Supplementary Table S4. Values are means ± SD over the five repeated splits; 95% confidence intervals were obtained by stratified bootstrap over test patients (1000 resamples) and averaged over the five splits.

## Data Availability

The cfDNA low-coverage whole-genome sequencing datasets analyzed in this study are publicly available. DELFI/Cristiano 2019 and Jiang 2015 data were obtained from the FinaleDB database (http://finaledb.research.cchmc.org). LUCAS/Mathios 2021 data were obtained from the European Genome-phenome Archive (https://ega-archive.org) under Dataset ID EGAD00001007796. The source code for FragMIL is publicly available at https://github.com/DrYong290526/FragMIL-Fragment-Token-Multiple-Instance-Learning-Framework (accessed on 18 October 2026) and the version used in this study (v1.0.0, MIT license) has been deposited in Zenodo at https://doi.org/10.5281/zenodo.21222682.

## Supplementary Materials

The following supporting information can be downloaded at https://www.mdpi.com/article/10.3390/cancers18203389/s1, Figure S1: Cancer detection precision–recall curves for fragment-token MIL and fragmentomic baseline feature groups across three cohorts; Figure S2: Fragment-token MIL AUPRC across fragment pools, branch modes, aggregation modules, and fragment budgets; Table S1: Sample-level cohort metadata; Table S2: Patient characteristics by class in the three cohorts; Table S3: Patient identifiers of the five repeated splits; Table S4: Per-repeat and mean AUROC and AUPRC of the four baseline feature groups for five classifiers (logistic regression, SVM, random forest, XGBoost, MLP) in the three cohorts; Table S5: Sensitivity, specificity, PPV and NPV at 90% and 95% target specificity for the best fragment-token MIL configuration and the strongest baseline; Table S6: Calibration of the FragMIL score on the test sets; Table S7: Top-3 best-performing fragment-token MIL configurations per cohort from the 288-configuration benchmark grid; Table S8: Full results of the 288-configuration benchmark grid; Table S9: Paired comparison of the best fragment-token MIL configuration with the strongest handcrafted baseline; Table S10: Subgroup AUROC with 95% confidence intervals and number of cancers per test split; Table S11: Performance of a single unified default FragMIL configuration, the cohort-specific post hoc optimal configurations and the strongest handcrafted baseline; Table S12: Enrichment of 5′ end motifs and short fragments among the 1% highest-attention fragment tokens in cancer cases correctly detected and non-cancer controls correctly classified by the unified default FragMIL configuration; Supplementary Methods: Data preprocessing, fragment-token encoding, fragment-pool and genomic-region definitions, MIL aggregator equations, baseline feature definitions and attention analysis.

## Abbreviations

The following abbreviations are used in this manuscript:

AUPRC	Area under the precision–recall curve
AUROC	Area under the receiver operating characteristic curve
cfDNA	Cell-free DNA
DELFI	DNA evaluation of fragments for early interception
FSD	Fragment-size distribution
HCC	Hepatocellular carcinoma
lcWGS	Low-coverage whole-genome sequencing
MIL	Multiple-instance learning
OCR	Open chromatin region
TSS	Transcription start site
