# Supplementary material for: FragMIL: A Fragment-Token Multiple-Instance Learning Framework for Cancer Detection from Cell-Free DNA Fragmentomics
Source: Cancers (Basel). 2026 Oct 20;18(20):3389. doi: 10.3390/cancers18203389 (PMC13702332; doi:10.3390/cancers18203389)
Supplement: Supplementary file 1 [file cancers-18-03389-s001.zip › Supplementary Figures S1 and S2.pdf]

Supplementary Materials

Supplementary Figures for: FragMIL: A Fragment-Token Multiple-Instance Learning Framework for Cancer Detection from Cell-Free DNA Fragmentomics

Tien-Dung Bui, Yung-Feng Lin, Mai-Hanh Nguyen, Shih-Feng Tsai, Nguyen-Quoc-Khanh Le * and Teh-Ying Chou *

* Correspondence: khanhlee@tmu.edu.tw (N.-Q.-K.L.); tehying@gmail.com (T.-Y.C.)

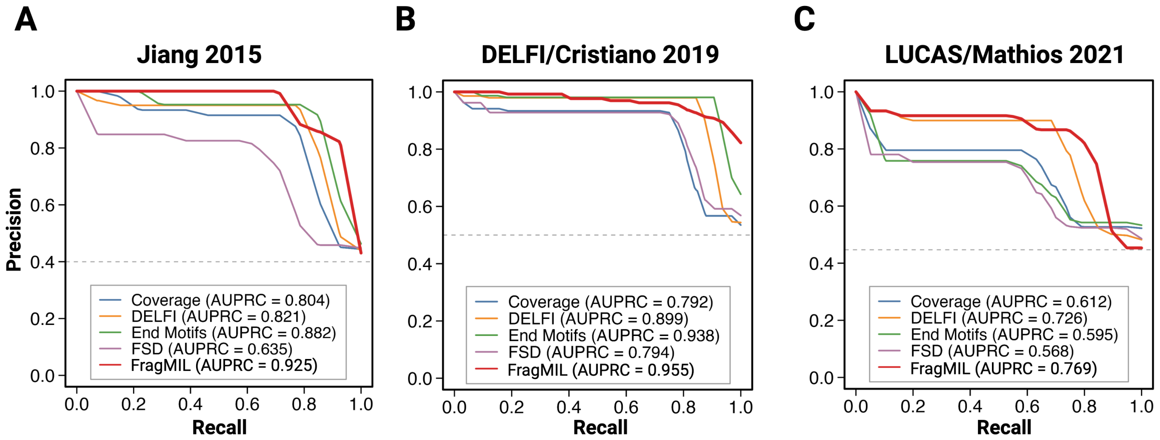

Figure S1. Cancer detection precision–recall curves for fragment-token MIL and fragmentomic baseline feature groups across three cohorts. Precision–recall curves comparing the best fragment-token MIL configuration with four established cfDNA fragmentomic features (Coverage, DELFI, End Motif, and Fragment size distribution, FSD) for (**A**) Jiang 2015 (HCC versus non-cancer controls), (**B**) DELFI/Cristiano 2019 (pan-cancer versus healthy controls), and (**C**) LUCAS/Mathios 2021 (lung cancer versus healthy controls). Dashed horizontal lines indicate the proportion of cancer samples in each cohort (no-skill baseline). AUPRC, area under the precision–recall curve; HCC, hepatocellular carcinoma.

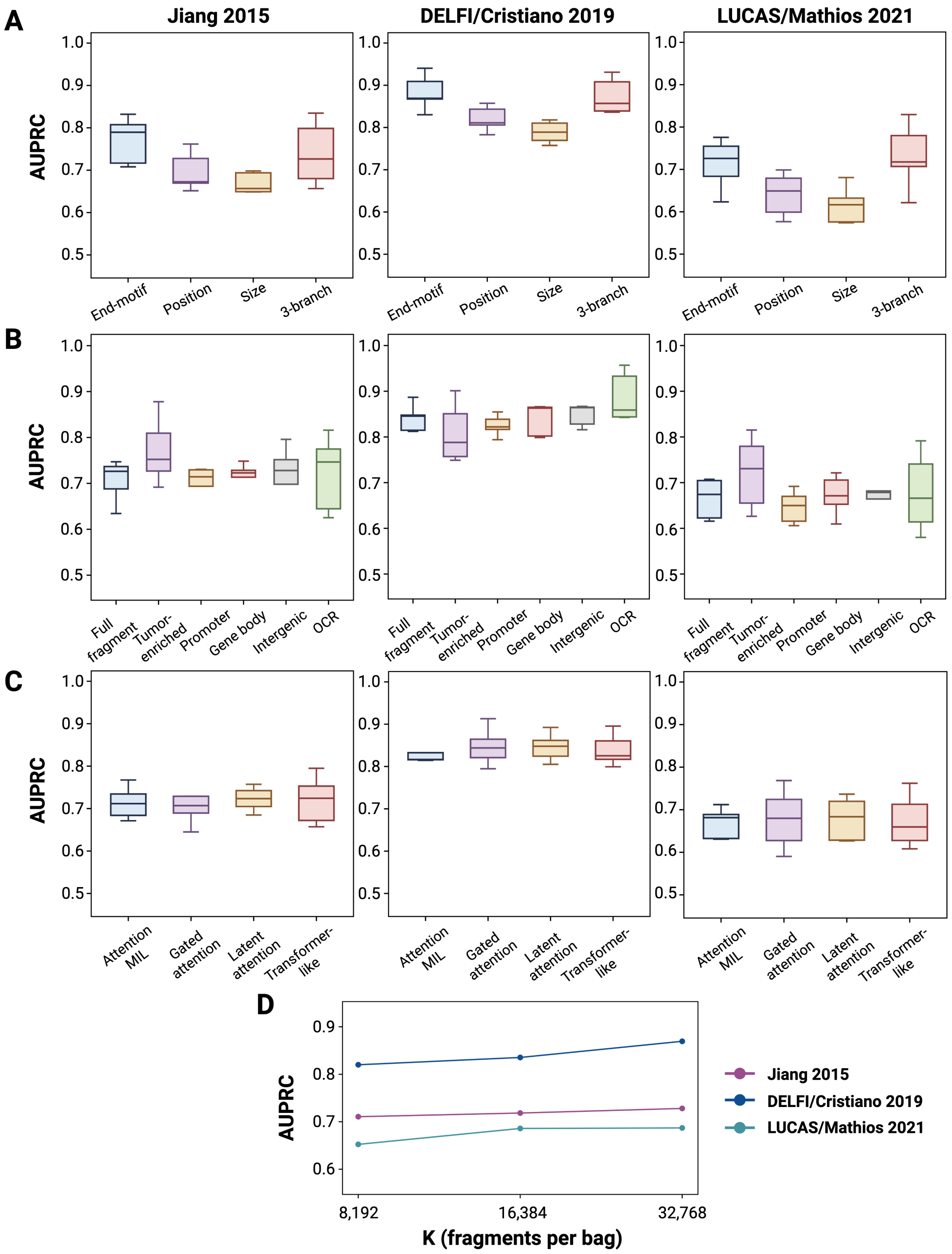

Figure S2. Fragment-token MIL AUPRC across fragment pools, branch modes, aggregation modules, and fragment budgets. (**A**) Branch mode (input branches used to encode each cfDNA fragment). (**B**) Fragment pool (genome-wide, tumor-enriched, or region-restricted cfDNA fragment subset). (**C**) Aggregation module (MIL architecture used to aggregate fragment embeddings into a patient-level score). (**D**) Fragment budget (K fragments sampled per patient bag at each training epoch). Box plots in (**A**–**C**) show marginal AUPRC across the five repeated subject-level splits; (**D**) shows mean marginal AUPRC for each fragment budget. AUPRC is the precision–recall counterpart of the AUROC analysis in Figure 3 of the main text. OCR, open chromatin regions.
