# Supplementary material for: FragMIL: A Fragment-Token Multiple-Instance Learning Framework for Cancer Detection from Cell-Free DNA Fragmentomics
Source: Cancers (Basel). 2026 Oct 20;18(20):3389. doi: 10.3390/cancers18203389 (PMC13702332; doi:10.3390/cancers18203389)
Supplement: Supplementary file 1 [file cancers-18-03389-s001.zip › Supplementary Methods.pdf]

Supplementary Materials

Supplementary Methods for: FragMIL: A Fragment-Token Multiple-Instance Learning Framework for Cancer Detection from Cell-Free DNA Fragmentomics

Tien-Dung Bui, Yung-Feng Lin, Mai-Hanh Nguyen, Shih-Feng Tsai, Nguyen-Quoc-Khanh Le * and Teh-Ying Chou *

* Correspondence: khanhlee@tmu.edu.tw (N.-Q.-K.L.); tehying@gmail.com (T.-Y.C.)

Contents: S1. Reprocessing of fragment BED files; S2. Fragment-token construction; S3. Genomic-region and fragment-pool definitions; S4. MIL aggregation equations; S5. Handcrafted fragmentomic baseline features; S6. Attention analysis of high-weight fragments; References.

S1. Reprocessing of fragment BED files

Fragment files of the DELFI/Cristiano 2019 and Jiang 2015 cohorts were obtained from FinaleDB [1], in which raw reads had been trimmed with Trimmomatic, aligned to hg19 with BWA-MEM and duplicate-marked with samblaster, and fragments had been inferred from properly paired, non-duplicate primary alignments. For the LUCAS/Mathios 2021 cohort, BAM files aligned to hg19 by the original investigators (Bowtie2, with PCR duplicates removed using Sambamba [2]) were obtained from the European Genome-phenome Archive and converted to fragment files in the same five-column format (chromosome, start, end, MAPQ, strand) with SAMtools: properly paired primary reads (SAM flags `-f 0x2 -F 0xB04`) with MAPQ $\geq$ 30 were retained, mates were paired by read name, and each fragment was defined by the outer alignment ends of the two mates, with the lower MAPQ of the two mates and the strand of read 1.

All analyses used the GRCh37/hg19 reference assembly. For each patient, the input was a fragment BED file containing inferred paired-end cfDNA fragments with fields: chromosome, start, end, mapQ/score, and strand. Fragment coordinates followed the standard 0-based half-open BED convention (start = 0-based inclusive; end = 0-based exclusive; fragment interval = [start, end)). For each fragment the following quantities were derived:

$$\begin{aligned} \mathit{fragment\_length} &= \mathit{end} - \mathit{start}, & \mathit{left\_end} &= \mathit{start}, \\ \mathit{right\_end} &= \mathit{end} - 1, & \mathit{midpoint} &= \left\lfloor \frac{\text{start+end}}{2} \right\rfloor. \end{aligned}$$

Only autosomal fragments on chromosomes chr1–chr22 were retained, with valid coordinates, positive length, valid strand annotation (+ or −), and MAPQ $\geq$ 30. Fragments with ambiguous reference bases within either 8-bp endpoint sequence were removed before token construction. PCR duplicates had been removed when the fragment files were generated from the aligned reads, and no additional deduplication was applied.

A unified bad-region mask was constructed from the UCSC hg19 assembly gap track and ENCODE blacklist v2 [3]. Gap and blacklist intervals were restricted to chr1–chr22, sorted, merged, and used to remove fragments overlapping problematic genomic regions. UCSC hg19 chromosome-size and centromere annotations were used to define chromosome boundaries, construct 1-Mb and 100-kb genomic bins, and assign each fragment midpoint to the p or q chromosome arm.

S2. Fragment-token construction

Each high-quality autosomal fragment was encoded as a fragment token consisting of three complementary branches: a paired-end motif branch, a genomic-position branch, and a fragment-length branch. The three branch embeddings were concatenated and fused to generate a fragment-level embedding.

S2.1. Paired-end motif branch

The 8-bp sequence at the left endpoint and the 8-bp sequence at the right endpoint were extracted from the reference genome. Each nucleotide was encoded as a four-dimensional one-hot vector:

$$\mathbf{A} = [1, 0, 0, 0], \quad \mathbf{C} = [0, 1, 0, 0], \quad \mathbf{G} = [0, 0, 1, 0], \quad \mathbf{T} = [0, 0, 0, 1].$$

For a patient bag containing K fragments, the left-end and right-end sequences were represented as tensors of shape $[K, 8, 4]$. The two endpoint tensors were each flattened to a 32-element vector (8 bases $\times$ 4), so that together 16 endpoint bases contributed 64 motif values per fragment. Fragment strand was encoded as a two-dimensional one-hot vector:

$$(+) \text{ strand} = [1, 0], \qquad (-) \text{ strand} = [0, 1].$$

The 64 endpoint motif values and the 2 strand values were concatenated to form a 66-dimensional motif input vector $\mathbf{m} \in \mathbb{R}^{66}$ for each fragment. Endpoint sequences were not reverse-complemented or swapped according to strand; strand identity was supplied directly as part of the motif input. A two-layer MLP with ReLU activation transformed $\mathbf{m}$ into a motif embedding $\mathbf{e}_{\text{motif}} \in \mathbb{R}^d$.

S2.2. Genomic-position branch

Four categorical variables were defined per fragment: chromosome identity (chr1–chr22), chromosome arm (p or q), 1-Mb genomic bin, and 100-kb genomic bin. Each categorical variable was mapped through a learnable embedding layer. To preserve local spatial context within each 100-kb bin, three normalized local coordinates were computed for the fragment start x_s, fragment end x_e, and fragment midpoint x_m. For a genomic coordinate x within a 100-kb bin $[b_{\text{start}}, b_{\text{end}})$, the normalized local coordinate was:

$$\tilde{x} = \frac{x - b_{\text{start}}}{b_{\text{end}} - b_{\text{start}}}, \qquad x \in \{x_s, x_e, x_m\}. \tag{S1}$$

The four categorical embeddings and the three normalized local coordinates were concatenated and passed through a position encoder MLP to produce a position embedding $\mathbf{e}_{\text{pos}} \in \mathbb{R}^d$.

S2.3. Fragment-length branch

For each patient, the mean μ_L and standard deviation σ_L of fragment length were computed from all high-quality autosomal fragments, independent of the fragment pool being analyzed. For a fragment of length L, the patient-normalized z-score was:

$$z_L = \frac{L - \mu_L}{\sigma_L}. \tag{S2}$$

This scalar z-score was passed through a size encoder MLP to produce a length embedding $\mathbf{e}_{\text{size}} \in \mathbb{R}^d$.

S2.4. Branch fusion

In the three-branch model, the motif, position, and size embeddings were concatenated along the feature dimension and passed through a fusion MLP with ReLU activation to produce the final fragment-level embedding $\mathbf{e}_{\text{token}} \in \mathbb{R}^d$:

$$\mathbf{e}_{\text{token}} = \text{FusionMLP}\big([\, \mathbf{e}_{\text{motif}} \,;\, \mathbf{e}_{\text{pos}} \,;\, \mathbf{e}_{\text{size}} \,]\big). \tag{S3}$$

In single-branch input modes, only the corresponding branch embedding was used; no fusion MLP was applied.

S3. Genomic-region and fragment-pool definitions

All genomic regions and fragment pools were defined in hg19 coordinates.

S3.1. Full-fragment pool

The Full-fragment pool included all high-quality autosomal fragments after preprocessing, representing the genome-wide random-fragment setting.

S3.2. Tumor-enriched fragment pool

The Tumor-enriched pool retained fragments satisfying both a size criterion and a nucleosome-overlap criterion. The size criterion retained fragments in either of the following windows: mono-nucleosomal tumor-enriched window (127–141 bp) or di-nucleosomal tumor-enriched window (272–292 bp), motivated by prior cfDNA fragment-size analysis showing that selected size ranges can enrich tumor-derived cfDNA signal [4,5]. The nucleosome-overlap criterion required at least one fragment endpoint to overlap a nucleosome-protected region. Nucleosome-protected regions were derived from the NucMap GM12878 DANPOS nucleosome peak set [6,7]. Peak centers were extended ±73 bp to obtain 147-bp nucleosome-protected windows (the canonical nucleosome core length). For each fragment, endpoint intervals were defined as:

$$\text{left endpoint interval} = [\text{start},\ \text{start} + 1), \quad \text{right endpoint interval} = [\text{end} - 1,\ \text{end}).$$

A fragment passed the nucleosome-overlap criterion if either endpoint interval overlapped a nucleosome-protected region. Endpoint–nucleosome overlap was used only for fragment-pool selection and was not supplied to the model as an input feature.

S3.3. Promoter regions

Promoter regions were defined as gene-level TSS ±500 bp windows using UCSC hg19 refGene annotation. For each gene, a representative TSS was selected from transcript annotations. Promoter windows were clipped to chromosome boundaries, restricted to chr1–chr22, and filtered against the bad-region mask. Promoter intervals were allowed to overlap gene-body intervals because TSS-centered windows can extend into the transcribed region. TSS-proximal cfDNA coverage and fragmentation patterns were included because previous studies showed that plasma DNA coverage near TSSs reflects gene-expression-related nucleosome organization [8].

S3.4. Gene body regions

Gene-body regions were defined by collapsing all transcripts assigned to each gene into a single gene-level interval from the minimum transcript start to the maximum transcript end. Gene-body intervals were restricted to chr1–chr22 and filtered against the bad-region mask.

S3.5. Intergenic regions

Intergenic regions were defined as the autosomal genome after removing promoter intervals, gene-body intervals, and the bad-region mask. Fragments assigned to the Intergenic pool did not overlap promoter or gene-body regions by midpoint.

S3.6. Open chromatin regions

Open chromatin regions (OCRs) were constructed by integrating Roadmap Epigenomics DNase hypersensitivity broad-peak tracks from blood-related references E029, E032, and E034 with the TCGA pan-cancer ATAC-seq peak set [9,10]. TCGA ATAC-seq peaks were converted to hg19 coordinates when required. All OCR intervals were restricted to chr1–chr22, merged, and standardized to fixed-width 200-bp windows centered on each merged peak centroid. OCR windows overlapping the bad-region mask were removed. OCR-based fragmentation was included because open chromatin regions carry tissue-of-origin and regulatory-region cfDNA fragmentation information [11].

S3.7. Fragment assignment to region pools

Fragments were assigned to region-specific pools using midpoint overlap. For each fragment, a 1-bp midpoint interval [midpoint, midpoint + 1) was intersected with each region set. Midpoint-based assignment reduces double-counting of long fragments spanning adjacent regions. Promoter and gene-body pools were not forced to be mutually exclusive, whereas the Intergenic pool was strictly non-overlapping with both promoter and gene-body regions. OCRs were treated as functional annotations and could overlap promoter, gene-body, or intergenic intervals.

S4. MIL aggregation equations

For each patient, the fragment encoder produced a bag of fragment embeddings $\mathbf{H} = \{\mathbf{h}_1, \ldots, \mathbf{h}_K\}$, $\mathbf{h}_k \in \mathbb{R}^d$. The MIL aggregation module transformed this unordered set into a patient embedding $\mathbf{z} \in \mathbb{R}^d$, which was then passed to a classifier MLP to generate a patient-level cancer probability.

S4.1. Attention MIL

Attention MIL [12] assigned a learnable scalar score to each fragment embedding:

$$\alpha_k = \mathbf{w}^\top \tanh(\mathbf{V}\mathbf{h}_k)\,, \tag{S4}$$

where $\mathbf{w} \in \mathbb{R}^L$ and $\mathbf{V} \in \mathbb{R}^{L\times d}$ are learnable parameters. Attention weights were normalized across all K fragments via softmax:

$$a_k = \frac{\exp(\alpha_k)}{\sum_{j=1}^{K} \exp(\alpha_j)}. \tag{S5}$$

The patient embedding was computed as the attention-weighted sum:

$$\mathbf{z} = \sum_{k=1}^{K} a_k\, \mathbf{h}_k. \tag{S6}$$

S4.2. Gated attention MIL

Gated attention MIL [12] used two parallel transformations before scalar attention scoring:

$$\alpha_k = \mathbf{w}^\top \Big[\tanh(\mathbf{V}\mathbf{h}_k) \odot \sigma(\mathbf{U}\mathbf{h}_k)\Big], \tag{S7}$$

where $\mathbf{w} \in \mathbb{R}^L$, $\mathbf{V} \in \mathbb{R}^{L\times d}$, and $\mathbf{U} \in \mathbb{R}^{L\times d}$ are learnable parameters, $\sigma(\cdot)$ is the sigmoid function, and $\odot$ denotes element-wise multiplication. Attention weights a_k and the patient embedding $\mathbf{z}$ were then computed using Equations (S5) and (S6).

S4.3. Latent attention

Latent attention [13] introduced M learnable latent vectors $\mathbf{L} = [\mathbf{l}_1, \ldots, \mathbf{l}_M]^\top \in \mathbb{R}^{M\times d}$. These latent vectors cross-attended to the fragment embeddings via scaled dot-product attention:

$$\mathbf{L}' = \mathrm{softmax}\left(\frac{\mathbf{L}\mathbf{W}_Q\,(\mathbf{H}\mathbf{W}_K)^\top}{\sqrt{d_k}}\right)\mathbf{H}\mathbf{W}_V, \tag{S8}$$

where $\mathbf{W}_Q, \mathbf{W}_K, \mathbf{W}_V \in \mathbb{R}^{d\times d_k}$ are learnable projection matrices. The resulting latent outputs $\mathbf{L}' \in \mathbb{R}^{M\times d_k}$ were mean-pooled to produce the patient embedding $\mathbf{z}$.

S4.4. Transformer-like latent attention

The transformer-like latent attention module first applied latent-to-fragment cross-attention as described in Equation (S8). The resulting latent vectors were then passed through self-attention among latent vectors, feed-forward layers, and residual connections [14]. The final latent representations were mean-pooled to generate the patient embedding $\mathbf{z}$. Full self-attention over all K fragment embeddings was not used because its memory complexity scales as $O(K^2)$, which is impractical for bags of 16,384 or 32,768 fragments.

S5. Handcrafted fragmentomic baseline features

Four established fragmentomic feature groups were used as baseline comparators: Coverage, DELFI, End Motif, and Fragment Size, selected to represent commonly used coverage-, genome-bin ratio-, end motif-, and size-based cfDNA fragmentomic signals [4,15–17].

S5.1. Coverage

Coverage features summarized cfDNA fragment coverage across promoter-proximal TSS windows. For each sample, fragments assigned to each TSS/promoter window were counted and normalized to form a patient-level coverage feature vector. This baseline was included because cfDNA coverage patterns near TSSs reflect nucleosome organization and gene-expression-related chromatin structure [8].

S5.2. DELFI

DELFI features were calculated from 5-Mb autosomal genomic bins following the genome-wide cfDNA fragmentation strategy introduced by Cristiano et al. [15]. Within each bin, fragments were separated into short and long size classes, and short-to-long fragment ratios were computed. GC correction was applied to reduce sequence-composition bias, and the corrected per-bin ratios formed the DELFI feature vector.

S5.3. End Motif

End Motif features summarized 5′ terminal 4-mer frequencies, following plasma DNA end-motif profiling approaches [16]. For each sample, all 256 possible 4-mer motifs were counted at fragment ends and normalized to frequencies f_i ($i = 1, \ldots, 256$). Motif diversity was calculated as normalized Shannon entropy:

$$H = -\frac{\sum_{i=1}^{256} f_i \log f_i}{\log 256}. \quad \text{(S9)}$$

The 256-dimensional 4-mer frequency vector and the motif diversity score H formed the End Motif feature group.

S5.4. Fragment Size

Fragment Size features summarized the fragment-length distribution from 75 to 400 bp. For each sample, fragment counts were tabulated by length and normalized to frequencies, generating a patient-level size-distribution vector. This baseline was included because fragment-size profiles and size-selected cfDNA fractions have been shown to enrich tumor-derived cfDNA signal and improve cancer detection [4,15].

Each baseline feature group was evaluated separately using L2-regularized logistic regression. Features were standardized using training-set statistics within each split, and the same five subject-level train/validation/test splits were used for baseline and fragment-token MIL evaluation.

S6. Attention analysis of high-weight fragments

The unified default configuration (Supplementary Table S11) was applied to the test patients of each split with 10 test-time bags per patient. Cancer cases correctly detected (true positives) and non-cancer controls correctly classified (true negatives) were defined at the operating point with 90% target specificity (Supplementary Table S5). In each bag, the 1% of fragments with the highest attention weight (328 of 32,768) were selected and pooled across bags, and compared with all fragments of the same bags. For each fragment end, the 5′ end motif was defined as the first four nucleotides read from the 5′ end (forward strand at the left end, reverse complement at the right end), and the proportions of CCCA and CCCG motifs and of fragments of 100–150 bp were computed. Fold enrichment (proportion among top-1% fragments divided by proportion among all fragments) was computed per patient, after averaging patients tested in more than one split. Fold enrichment was compared with 1 by the Wilcoxon signed-rank test of log fold enrichment and between true positives and true negatives by the Mann–Whitney test, with Holm adjustment across the nine comparisons (Supplementary Table S12).

References

1. Zheng, H.; Zhu, M.S.; Liu, Y. FinaleDB: A browser and database of cell-free DNA fragmentation patterns. *Bioinformatics* **2021**, *37*, 2502–2503. https://doi.org/10.1093/bioinformatics/btaa999
2. Mathios, D.; Johansen, J.S.; Cristiano, S.; Medina, J.E.; Phallen, J.; Larsen, K.R.; Bruhm, D.C.; Niknafs, N.; Ferreira, L.; Adleff, V.; et al. Detection and characterization of lung cancer using cell-free DNA fragmentomes. *Nat. Commun.* **2021**, *12*, 5060. https://doi.org/10.1038/s41467-021-24994-w
3. Amemiya, H.M.; Kundaje, A.; Boyle, A.P. The ENCODE blacklist: Identification of problematic regions of the genome. *Sci. Rep.* **2019**, *9*, 9354. https://doi.org/10.1038/s41598-019-45839-z
4. Mouliere, F.; Chandrananda, D.; Piskorz, A.M.; Moore, E.K.; Morris, J.; Ahlborn, L.B.; Mair, R.; Goranova, T.; Marass, F.; Heider, K.; et al. Enhanced detection of circulating tumor DNA by fragment size analysis. *Sci. Transl. Med.* **2018**, *10*, eaat4921. https://doi.org/10.1126/scitranslmed.aat4921
5. Marass, F.; Stephens, D.; Ptashkin, R.; Zehir, A.; Berger, M.F.; Solit, D.B.; Diaz, L.A.; Tsui, D.W.Y. Fragment size analysis may distinguish clonal hematopoiesis from tumor-derived mutations in cell-free DNA. *Clin. Chem.* **2020**, *66*, 616–618. https://doi.org/10.1093/clinchem/hvaa026

6. Zhao, Y.; Wang, J.; Liang, F.; Liu, Y.; Wang, Q.; Zhang, H.; Jiang, M.; Zhang, Z.; Zhao, W.; Bao, Y.; et al. NucMap: A database of genome-wide nucleosome positioning map across species. *Nucleic Acids Res.* **2019**, *47*, D163–D169. https://doi.org/10.1093/nar/gky980
7. Chen, K.; Xi, Y.; Pan, X.; Li, Z.; Kaestner, K.; Tyler, J.; Dent, S.; He, X.; Li, W. DANPOS: Dynamic analysis of nucleosome position and occupancy by sequencing. *Genome Res.* **2013**, *23*, 341–351. https://doi.org/10.1101/gr.142067.112
8. Ulz, P.; Thallinger, G.G.; Auer, M.; Graf, R.; Kashofer, K.; Jahn, S.W.; Abete, L.; Pristauz, G.; Petru, E.; Geigl, J.B.; et al. Inferring expressed genes by whole-genome sequencing of plasma DNA. *Nat. Genet.* **2016**, *48*, 1273–1278. https://doi.org/10.1038/ng.3648
9. Roadmap Epigenomics Consortium; Kundaje, A.; Meuleman, W.; Ernst, J.; Bilenky, M.; Yen, A.; Heravi-Moussavi, A.; Kheradpour, P.; Zhang, Z.; Wang, J.; et al. Integrative analysis of 111 reference human epigenomes. *Nature* **2015**, *518*, 317–330. https://doi.org/10.1038/nature14248
10. Corces, M.R.; Granja, J.M.; Shams, S.; Louie, B.H.; Seoane, J.A.; Zhou, W.; Silva, T.C.; Groeneveld, C.; Wong, C.K.; Cho, S.W.; et al. The chromatin accessibility landscape of primary human cancers. *Science* **2018**, *362*, eaav1898. https://doi.org/10.1126/science.aav1898
11. Sun, K.; Jiang, P.; Cheng, S.H.; Cheng, T.H.T.; Wong, J.; Wong, V.W.S.; Ng, S.S.M.; Ma, B.B.Y.; Leung, T.Y.; Chan, S.L.; et al. Orientation-aware plasma cell-free DNA fragmentation analysis in open chromatin regions informs tissue of origin. *Genome Res.* **2019**, *29*, 418–427. https://doi.org/10.1101/gr.242719.118
12. Ilse, M.; Tomczak, J.M.; Welling, M. Attention-based deep multiple instance learning. In Proceedings of the 35th International Conference on Machine Learning, Stockholm, Sweden, 10–15 July 2018; Dy, J., Krause, A., Eds.; PMLR: **2018**; pp. 2127–2136.
13. Lee, J.; Lee, Y.; Kim, J.; Kosiorek, A.; Choi, S.; Teh, Y.W. Set Transformer: A framework for attention-based permutation-invariant neural networks. In Proceedings of the 36th International Conference on Machine Learning, Long Beach, CA, USA, 9–15 June 2019; PMLR: **2019**; pp. 3744–3753.
14. Vaswani, A.; Shazeer, N.; Parmar, N.; Uszkoreit, J.; Jones, L.; Gomez, A.N.; Kaiser, Ł.; Polosukhin, I. Attention is all you need. In Advances in Neural Information Processing Systems 30 (NeurIPS 2017); Curran Associates: Red Hook, NY, USA, **2017**; pp. 5998–6008.
15. Cristiano, S.; Leal, A.; Phallen, J.; Fiksel, J.; Adleff, V.; Bruhm, D.C.; Jensen, S.Ø.; Medina, J.E.; Hruban, C.; White, J.R.; et al. Genome-wide cell-free DNA fragmentation in patients with cancer. *Nature* **2019**, *570*, 385–389. https://doi.org/10.1038/s41586-019-1272-6
16. Jiang, P.; Sun, K.; Peng, W.; Cheng, S.H.; Ni, M.; Yeung, P.C.; Heung, M.M.S.; Xie, T.; Shang, H.; Zhou, Z.; et al. Plasma DNA end-motif profiling as a fragmentomic marker in cancer, pregnancy, and transplantation. *Cancer Discov.* **2020**, *10*, 664–673. https://doi.org/10.1158/2159-8290.CD-19-0622
17. Hou, Y.; Meng, X.; Zhou, X. Systematically evaluating cell-free DNA fragmentation patterns for cancer diagnosis and enhanced cancer detection via integrating multiple fragmentation patterns. *Adv. Sci.* **2024**, *11*, 2308243. https://doi.org/10.1002/advs.202308243
