# Supplementary material for: FragMIL: A Fragment-Token Multiple-Instance Learning Framework for Cancer Detection from Cell-Free DNA Fragmentomics
Source: Cancers (Basel). 2026 Oct 20;18(20):3389. doi: 10.3390/cancers18203389 (PMC13702332; doi:10.3390/cancers18203389)
Supplement: Supplementary file 1 [file cancers-18-03389-s001.zip › Table S7.pdf]

Supplementary Table S7

Supplementary Table S7. Top-3 best-performing fragment-token MIL configurations per cohort from the 288-configuration benchmark grid.

Rank	Cohort	Fragment pool	K	Branch mode	MIL aggregator	AUROC, mean ± SD	AUPRC, mean ± SD
**1**	DELFI/Cristiano 2019	**OCR**	**32,768**	**All three**	**Gated attention MIL**	**0.963 ± 0.009**	**0.955 ± 0.015**
2		OCR	32,768	End motif	Gated attention MIL	0.945 ± 0.038	0.957 ± 0.027
3		OCR	32,768	End motif	Latent attention	0.942 ± 0.045	0.953 ± 0.034
**1**	LUCAS/Mathios 2021	**Tumor-enriched**	**8,192**	**End motif**	**Latent attention**	**0.802 ± 0.009**	**0.769 ± 0.016**
2		Tumor-enriched	32,768	All three	Attention MIL	0.772 ± 0.161	0.781 ± 0.122
3		Tumor-enriched	16,384	All three	Gated attention MIL	0.771 ± 0.161	0.793 ± 0.125
**1**	Jiang 2015	**Tumor-enriched**	**32,768**	**End motif**	**Latent attention**	**0.910 ± 0.015**	**0.925 ± 0.008**
2		Tumor-enriched	32,768	End motif	Gated attention MIL	0.857 ± 0.097	0.844 ± 0.094
3		Tumor-enriched	32,768	End motif	Attention MIL	0.855 ± 0.096	0.839 ± 0.092

Configurations are ranked by AUROC mean across five independent subject-level splits (70/15/15 train/validation/test). Shaded rows (Rank 1) correspond to the best-performing configuration reported in Table 2 of the main text. Fragment pools — OCR: open chromatin regions (200-bp fixed-width windows centered on merged peak centroids); Tumor-enriched: mono-nucleosomal (127–141 bp) or di-nucleosomal (272–292 bp) fragments with at least one endpoint overlapping a nucleosome-protected region; Full: all high-quality autosomal fragments. Branch modes — All three: paired-end motif, genomic position, and patient-normalized fragment length; End motif: paired-end motif branch only. K, number of fragment tokens sampled per patient bag per training epoch. AUROC, area under the receiver operating characteristic curve; AUPRC, area under the precision–recall curve.
